# Prevention of Anal Condyloma with Quadrivalent Human Papillomavirus Vaccination of Older Men Who Have Sex with Men

**DOI:** 10.1371/journal.pone.0093393

**Published:** 2014-04-08

**Authors:** Kristin A. Swedish, Stephen E. Goldstone

**Affiliations:** 1 Department of Internal Medicine, Montefiore Medical Center, Bronx, New York, United States of America; 2 Department of Surgery, Icahn School of Medicine at Mount Sinai, New York, New York, United States of America; State University of Maringá/Universidade Estadual de Maringá, Brazil

## Abstract

**Background:**

The quadrivalent human papillomavirus vaccine (qHPV) is FDA-approved for use in males 9 to 26 years old to prevent anogenital condyloma. The objective of this study is to determine if qHPV is effective at preventing anal condyloma among men who have sex with men (MSM) aged 26 years and older.

**Methods:**

This post-hoc analysis of a nonconcurrent cohort study evaluated 210 patients without history of anal condyloma and 103 patients with previously-treated anal condyloma recurrence-free for at least 12 months prior to vaccination/time zero. We determined the rate of anal condyloma development in vaccinated versus unvaccinated patients.

**Results:**

313 patients with mean age 42 years were followed for median 981 days. During 773.6 person-years follow-up, condyloma developed in 10 (8.6%) vaccinated patients (incidence of 3.7 per 100 person-years) and 37 (18.8%) unvaccinated patients (incidence 7.3 per 100 person-years; p = 0.05). Multivariable hazards ratio showed that qHPV was associated with decreased risk of anal condyloma development (HR 0.45; 95% CI 0.22–0.92; p = 0.03). History of anal condyloma was associated with increased risk of anal condyloma development (HR 2.28; 95% CI 1.28–4.05; p = 0.005), as was infection with oncogenic HPV (HR 3.87; 95% CI 1.66–9.03; p = 0.002).

**Conclusions:**

Among MSM 26 years of age and older with and without history of anal condyloma, qHPV reduces the risk of anal condyloma development. A randomized controlled trial is needed to confirm these findings in this age group.

## Introduction

Anogenital condyloma affect approximately 1% of the American sexually active population at any one time[1] and are more prevalent among men who have sex with men (MSM) than among men who have sex with women. A study of over 2000 men aged 16 to 26 found the incidence rate of anogenital condyloma was more than three times higher among MSM than men who identified as heterosexual (4.7 per 100 person-years vs. 1.5 per 100 person-years; unpublished data). An international study of men aged 18 to 70 found that men with 3 or more male anal sex partners in the past 3 months were 4.5 times more likely than men with no male partners to develop anogenital condyloma[2].

The quadrivalent HPV vaccine (qHPV; Gardasil, Merck & Co., Inc, Whitehouse Station, NJ) protects against the 4 most common HPV types, including non-oncogenic types HPV 6 and 11, which account for approximately 90% of all anogenital condyloma[3]. In their study of qHPV among young men aged 16 to 26 without history of anogenital condyloma, Giuliano et al. found that qHPV was 89% effective against condyloma development in those PCR and seronegative for the 4 qHPV types at baseline. However, the vaccine was shown to be more effective among heterosexual males (92% efficacy) than MSM (79% efficacy; against external genital lesions, the majority of which were condyloma). In the intention-to-treat population (heterosexual males and MSM who may not have received all 3 doses of qHPV and/or may have been PCR or seropositive for any of the 4 qHPV types at baseline), efficacy to prevent condyloma dropped to 67% [4]. Based upon the results of this clinical trial, the United States Food and Drug Administration licensed qHPV for use in males 9 to 26 years old to prevent anogenital condyloma caused by HPV 6 and 11[5]. In a substudy of just MSM aged 16–26, qHPV demonstrated 100% efficacy preventing intra-anal condyloma in subjects PCR and seronegative for the 4 qHPV types and 57.2% in the intention to treat population[6]. Further analysis limited to men who were seronegative for the 4 qHPV types and PCR-negative to 10 non-qHPV types found 85% efficacy of qHPV against condyloma. In the intention-to-treat population (men who may not have received all 3 doses of qHPV and/or may have been PCR or seropositive for any of the 14 HPV types at baseline), qHPV efficacy against external genital lesions decreased to 59%[7].

Although the incidence and prevalence of anogenital condyloma in men decreases with age[2], [8], [9], older MSM remain at risk for their development. Although not associated with increased mortality, condyloma are a source of emotional distress and affect quality of life[10]. Treatment is expensive, estimated to cost over $800 per incident case in the United States[11]. Recurrence, defined as reappearance of condyloma within 12 months after complete clearance, ranges from 4% to 50%, depending on treatment modality and immune competence[12], [13]. In one private practice (SEG), all HIV-negative MSM were offered qHPV off-label regardless of age, history of anogenital condyloma, history of anal high-grade squamous intraepithelial lesion (HSIL), or prior HPV infection. Insurance typically did not cover the cost of the vaccine; many patients paid out of pocket. The following is a non-concurrent observational cohort study evaluating the effectiveness of qHPV in preventing anal condyloma among HIV-negative MSM patients 26 years of age and older in this practice.

## Methods

### Study Population and Data Collection

The methodology of this study has been reported elsewhere[14]. Briefly, study participants were recruited from a single anorectal surgery practice (SEG) in New York City that specializes in screening, diagnosis, and treatment of anorectal diseases. The majority of patients are MSM presenting with HPV related disease. Beginning in June 2006, the three-dose qHPV series was offered off-label to all HIV-negative MSM patients at each clinical visit.

This post-hoc analysis included patients who were 26 years of age or older, HIV-negative, and self-identified MSM. Patients were included if they had no prior history of anal condyloma or if they had previously-treated anal condyloma and were recurrence-free for at least 12 months prior to study entry. Medical charts of all eligible patients seen during 2007–2010 were screened for inclusion. Study patients were identified as exposed to vaccination when billing records found payment for all 3 qHPV doses and the medical record noted vaccination. Study patients were identified as unvaccinated when billing records showed no qHPV doses and the medical record did not indicate vaccination. Patients who did not receive all 3 doses of the vaccine or who were vaccinated elsewhere were excluded. As with our prior study, vaccinated patients entered the study 1 month after their third dose of qHPV (month 7). Non-vaccinated patients entered the study at least 7 months after their first practice visit[14].

In the practice, patients are typically evaluated for HPV-related disease with digital anorectal exam, standard anoscopy, anal cytology, and tested for the presence of oncogenic HPV using Hybrid-Capture 2 High-Risk HPV DNA Test (Digene Corporation, Gaithersburg, MD) when insurance coverage permitted. Any abnormality was evaluated further with high-resolution anoscopy (HRA; essentially colposcopy of the anal canal)[15]. Anal condyloma were diagnosed clinically when visualized and/or by pathologist review of biopsy from a lesion in the peri- or intra-anal region. In addition to abstracting qHPV history, clinical charts were reviewed for demographics, smoking status, sexually transmitted infections, history of anal condyloma, history of HSIL, and oncogenic HPV status. Patients with anal or penile condyloma at study start were excluded. Patients were followed for up to 4 years following study entry.

This study was approved by the Icahn School of Medicine at Mount Sinai Institutional Review Board. Informed consent was waived, as the data studied had already been collected for medical purposes. No funding was received.

### Statistical Analysis

The vaccinated and unvaccinated groups were compared on baseline demographics, smoking status, oncogenic HPV status, and history of STIs. Comparisons were done using Chi-Square tests for categorical variables; Fishers Exact test for categorical variables, as appropriate; Student’s T-tests for normally-distributed continuous variables; and Mann-Whitney U test for nonparametric continuous variables.

To determine effect of vaccine on anal condyloma development, Kaplan-Meier and Cox Proportional Hazards analysis compared time to anal condyloma diagnosis in vaccinated and unvaccinated study patients. For Kaplan-Meier, the Log-Rank test determined significance. For Cox Proportional Hazards, vaccination status, demographic characteristics, smoking status, history of anogenital condyloma, oncogenic HPV infection, and STIs were evaluated individually to identify variables associated with time to anal condyloma development. STIs diagnosed following study entry were treated as time-dependent variables. Variables with p-value ≤0.25 in individual analysis were evaluated in multivariable Cox Proportional Hazards analysis to identify those variables significantly associated with time to anal condyloma development. SPSS Version 19 (IBM Corporation, Somers, NY) was used for the analysis. A p-value of ≤0.05 was considered significant.

## Results

There were 694 HIV-negative MSM seen in the practice from 2007 to 2010. Based on chart review, 313 patients were 26 years of age or older without history of anal condyloma or with history of previously-treated anal condyloma recurrence-free for at least 12 months. Their mean age was 42.1 years with standard deviation (SD) 9.8, range 26.1 to 76.0 years. Data on race/ethnicity were missing for 25% (77 of 313) of study patients. Of those whose race/ethnicity was identified, 89% (210 of 236) were white. One hundred three patients had history of anal condyloma within 5 years of study entry, all of whom were recurrence-free for at least 12 months prior to study entry.

Of 313 eligible participants, 116 (37%) were vaccinated and 197 (63%) were unvaccinated. Vaccinated patients were significantly younger than unvaccinated patients (vaccinated mean age 38.6 years with SD 7.4, unvaccinated mean age 44.3 years with SD 10.3, p<0.001). Vaccinated patients were more likely to smoke cigarettes (21.6% vs 13.2%, p = 0.05). The groups were comparable in terms of history of anal condyloma, history of HSIL, oncogenic HPV status, and history of STIs (Table 1). Unvaccinated patients had longer follow-up time (median 1039 days compared with 880 days; p = 0.07). Rates of gonorrhea, chlamydia, and syphilis after study entry were comparable between groups (0–4%).

**Table 1:** Baseline characteristics of vaccinated and unvaccinated MSM, New York City, April 2007– January 2013 (N = 313) (Number [%]).

Characteristic		Vaccinated (n = 116)	Unvaccinated (n = 197)	p-value
Demographics				
Age (mean [range])		38.6 (26.1, 55.2)	44.3 (26.1, 76.0)	<0.001
Race/Ethnicity	White	73 (63)	137 (70)	0.14
	Black	1 (1)	6 (3)	
	Asian	7 (6)	4 (2)	
	Hispanic	2 (2)	6 (3)	
	Unknown	33 (28)	44 (22)	
Insurance status	None	8 (7)	18 (9)	0.08
	Public	1 (1)	11 (6)	
	Commercial	107 (92)	167 (85)	
Cigarette smoking	Smokers	25 (22)	26 (13)	0.05
	Non-smokers	91 (78)	171 (87)	
**Medical History**				
History of anogenital condyloma within five years prior to study entry	Yes	41 (35)	62 (32)	0.48
	No	75 (65)	135 (68)	
History of HSIL prior to study entry	Yes	52 (45)	77 (39)	0.32
	No	64 (55)	120 (61)	
Oncogenic HPV status^a^	Uninfected	43 (37)	68 (35)	0.08
	Infected	49 (42)	66 (34)	
	Unknown	24 (21)	63 (32)	
History of Gonorrhea	Yes	23 (20)	51 (26)	0.22
	No	93 (80)	146 (74)	
History of Chlamydia	Yes	18 (16)	18 (9)	0.09
	No	98 (84)	179 (91)	
History of Syphilis	Yes	3 (3)	10 (5)	0.22
	No	113 (97)	187 (95)	

a Oncogenic HPV status: within eight months prior to first vaccine dose (vaccinated patients) or within eight months prior to start time (unvaccinated patients).

Among vaccinated patients, 10 developed anal condyloma during 269.3 person-years of follow-up giving an incidence rate of 3.7 per 100 person-years (95% confidence interval [CI] 1.8–6.8/100 person-years). Among unvaccinated patients, 37 developed anal condyloma during 504.3 person-years of follow-up giving an incidence rate of 7.3 per 100 person-years (95% CI 5.2–10.1/100 person-years). The difference in incidence rates is significant (p = 0.05).

**Figure 1:**
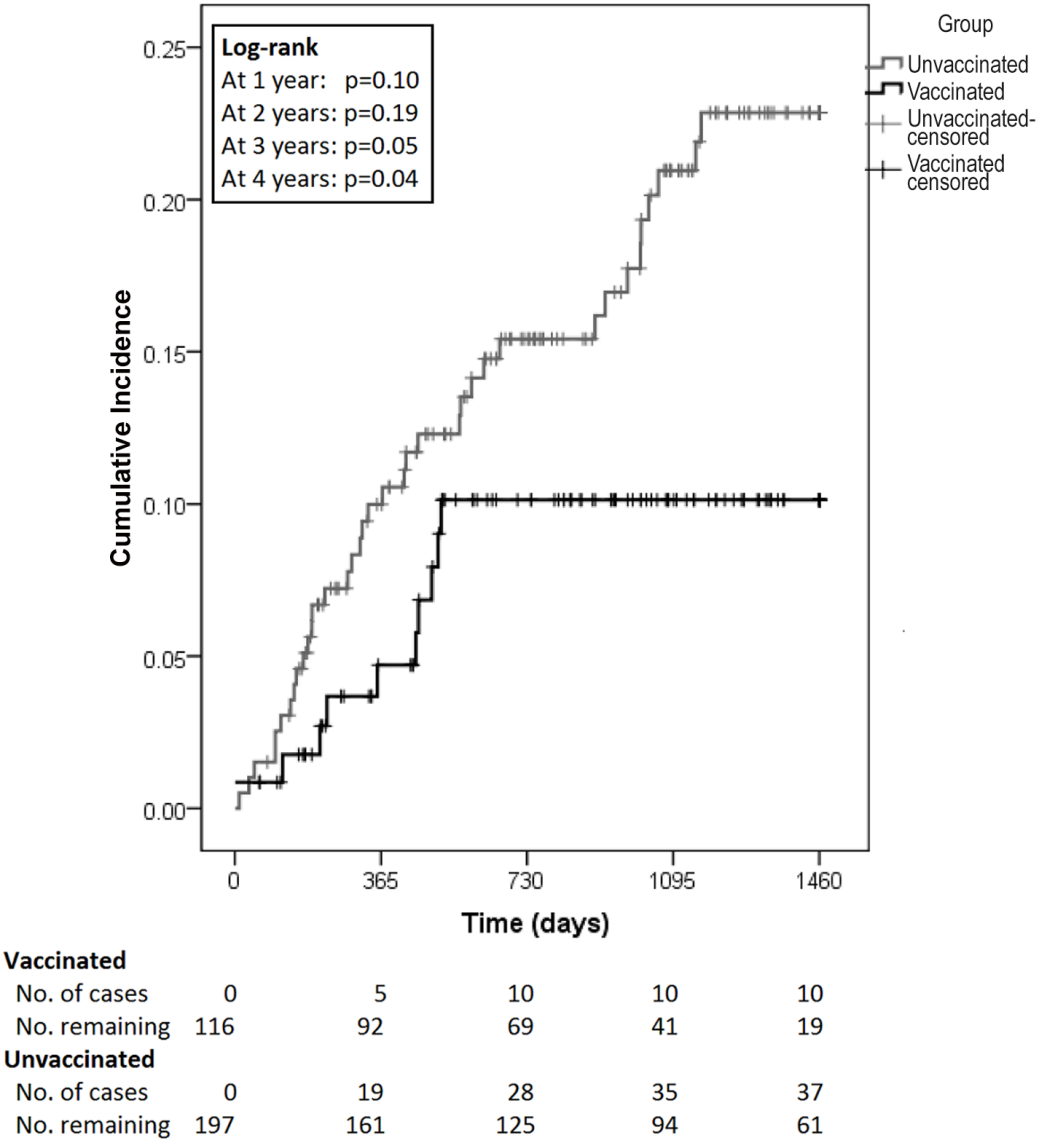
Time to anal condyloma development among vaccinated and unvaccinated MSM, New York City, April 2007– January 2013 (N = 313).

Kaplan-Meier survival analysis demonstrated improved condyloma-free survival of vaccinated persons compared with unvaccinated (log-rank p-value = 0.04; Figure 1). In univariate Cox proportional hazards analysis, qHPV vaccination was associated with decreased risk of developing anal condyloma (HR 0.49; 95% CI 0.24–0.98; p = 0.04). History of prior anal condyloma within five years was associated with increased risk of developing anal condyloma (HR 2.25; 95% CI 1.27–4.00; p = 0.005), as was infection with oncogenic HPV (HR 3.68; 95% CI 1.58–8.58; p = 0.003; Table 2). Multivariate Cox proportional hazards analysis showed that qHPV vaccination was associated with decreased risk of developing anal condyloma (HR 0.45; 95% CI 0.22–0.92; p = 0.03; Table 2). History of anal condyloma was associated with increased risk of developing anal condyloma (HR 2.28; 95% CI 1.28–4.05; p = 0.005), as was infection with oncogenic HPV (HR 3.87; 95% CI 1.66–9.03; p = 0.002).

**Table 2:** Characteristics associated with anal condyloma development among 313 MSM using Cox Proportional univariate and multivariate analysis, New York City, April 2007– January 2013.

Predictor Variable		Condyloma development (n = 47)	No condyloma development (n = 266)	p-value	Univariate HR (95% CI)	p-value	Multivariate HR (95% CI)	p-value
qHPV vaccine status	Vaccinated	10 (21)	106 (40)	0.02	0.49 (0.24, 0.98)	0.04	0.45 (0.22, 0.92)	0.03
	Unvaccinated	37 (79)	160 (60)					
Age (mean [SD])		44.1 (9.3)	41.8 (9.9)	0.13	1.02 (0.99, 1.05)	0.24		
Race	White	33 (70)	177 (67)	0.83	1.00			
	Black	1 (2)	6 (2)		0.84 (0.12, 6.14)	0.86		
	Hispanic	2 (4)	9 (3)		1.43 (0.34, 5.97)	0.62		
	Asian	2 (4)	6 (2)		1.74 (0.42, 7.27)	0.45		
	Unknown	9 (19)	68 (26)		0.85 (0.41, 1.78)	0.67		
Insurance	None	4 (9)	22 (8)	0.80	0.88 (0.32, 2.45)	0.80		
	Public	1 (2)	11 (4)		0.51 (0.07, 3.72)	0.51		
	Commercial	42 (89)	232 (88)		1.00			
Cigarette smoking	Yes	8 (17)	43 (16)	0.88	1.12 (0.53, 2.41)	0.76		
	No	39 (83)	223 (84)					
Anogenital condyloma within 5 years prior to study entry	Yes	25 (53)	78 (29)	0.001	2.25 (1.27, 4.00)	0.005	2.28 (1.28, 4.05)	0.005
	No	22 (47)	188 (71)					
History of HSIL prior to study entry	Yes	21 (45)	108 (41)	0.60	1.22 (0.69, 2.17)	0.49		
	No	26 (55)	158 (59)					
Oncogenic HPV infection prior to time zero	Uninfected	7 (15)	104 (39)	0.006	1.00		1.00	
	Infected	23 (49)	92 (35)		3.68 (1.58, 8.58)	0.003	3.87 (1.66, 9.03)	0.002
	Unknown	17 (36)	70 (26)		3.30 (1.37, 7.95)	0.008	2.79 (1.15, 6.76)	0.02
Gonorrhea infection following study entry	Yes	2 (4)	10 (4)	0.56	1.00 (ND^a^)	1.00		
	No	45 (96)	256 (96)					
Chlamydia infection following study entry	Yes	3 (6)	7 (3)	0.18	1.00 (ND^a^)	1.00		
	No	44 (94)	259 (97)					
Syphilis infection following study entry	Yes	2 (4)	3 (1)	0.16	1.00 (ND^a^)	1.00		
	No	45 (96)	363 (99)					

a ND  =  not defined.

Of 103 patients with history of anogenital condyloma within five years of study entry, 41 (40%) patients were vaccinated and 62 (60%) were unvaccinated. Among vaccinated patients, 6 developed anal condyloma during 100.6 person-years of follow-up giving an incidence rate of 6.0 per 100 person-years (95% CI 2.2–13.0/100 person-years). Among unvaccinated patients, 19 developed anal condyloma during 158.7 person-years of follow-up giving an incidence rate of 12.0 per 100 person-years (95% CI 7.2–18.7/100 person-years). The difference in incidence rates was not significant (p = 0.13).

**Figure 2:**
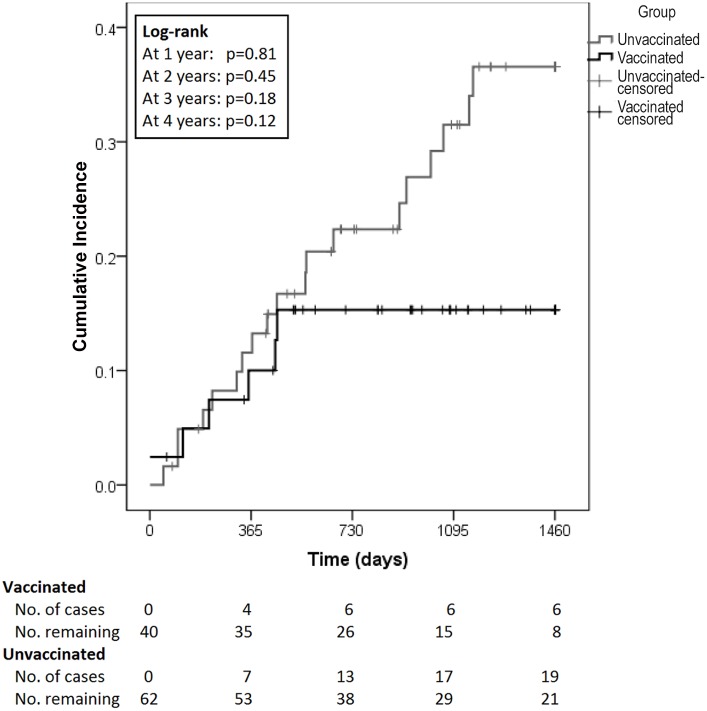
Time to recurrent anal condyloma development among vaccinated and unvaccinated MSM with history of anogenital condyloma, New York City, April 2007– January 2013 (N = 103).

Of those patients with history of anogenital condyloma, Kaplan-Meier survival analysis demonstrated improved but insignificant condyloma-free survival of vaccinated persons compared with unvaccinated persons (log-rank p = 0.12; Figure 2).

## Discussion

This is the first study to examine the efficacy of qHPV against anal condyloma among older MSM. Older MSM are more likely to have been exposed to HPV in the past, as evidenced by our finding that over half of those tested were positive for oncogenic HPV in the 8 months prior to study entry. Commercial tests typically do not evaluate for non-oncogenic HPV types, though it is likely that patients exposed to oncogenic HPV may have also been exposed to the non-oncogenic HPV types that cause condyloma. A study of over 1200 HIV-negative MSM found 26% prevalence of non-oncogenic HPV types and 26% prevalence of oncogenic HPV types within the anal canal across all age groups[16].

QHPV vaccine demonstrated 70% efficacy against external anogenital condyloma and 57% against intra-anal condyloma among MSM 26 years old and younger in the intention-to-treat population[4], [6]. While this population is comparable to ours in some respects, there are some major dissimilarities. Even though the participants in the intention-to-treat population may have been infected with HPV at outset, they had very few lifetime sexual partners (≤5). Moreover, although men enrolled in that study might have been carrying asymptomatic HPV, they were excluded if they had HPV-related disease prior to study entry. In contrast, patients seen in our practice often present with HPV-related complaints, as evidenced by one-third of patients in our study having prior history of condyloma. Although we do not have the data, our patients are also probably more likely to have had more sexual partners given their ages, with greater probability of previous or current infection with one or more HPV types. Even so, our study found vaccine efficacy of 54% against anal condyloma among these older, high-risk MSM, and is comparable to prior reports [4], [6], [7]. MSM 26 years of age and older who received qHPV had decreased risk of developing anal condyloma compared to those who did not receive the vaccine. However, the Kaplan-Meier curves did not significantly separate until 3 years following vaccination. A similar delayed effect was seen by Giuliano, et al., wherein the Kaplan-Meier curves for development of external genital lesions related to the four qHPV types did not separate significantly until 24 months in the intent-to-treat population. In the per-protocol population, the curves did not show significant separation until 30 months (log-rank p-values not published)[4]. Our findings, though on a much smaller scale, are comparable. A larger, prospective, randomized study of older MSM would be beneficial to elucidate the true time course of vaccine efficacy.

The delay in vaccine efficacy may be related to the vaccine itself or to as-yet undetermined factors related to the biology of non-oncogenic HPV. In our practice, flat, low-grade squamous intraepithelial (LSIL) lesions are not treated unless they grossly appear to be condyloma, a morphologic diagnosis rather than one based upon histology. While some untreated LSIL may fully regress or even progress to HSIL, some untreated LSIL could eventually develop into condyloma. These condyloma would then appear to be new disease, rather than evolution of disease present at study entry. In our prior report, we documented significant prevention of recurrent HSIL at 1 year following qHPV[14]. The earlier onset of response to vaccine for HSIL but not condyloma could result from our practice to eradicate all HSIL at each treatment, likely leaving far less occult disease behind. This would diminish the likelihood of recurrence due to missed lesions present at study entry for HSIL but not condyloma.

It is unfortunately that we were unable to test for the presence of non-oncogenic HPV. We did, however, find that infection with oncogenic HPV significantly increased the hazard of developing anal condyloma. Although condyloma are associated with non-oncogenic HPV types (6 and11), there are possible explanations for the finding of increased risk in the presence of oncogenic HPV infection. A synergistic relationship between oncogenic and non-oncogenic HPV infections is possible, as evidence by the fact that women infected intra-anally with multiple HPV types were more likely to acquire additional infections and more likely to clear HPV infection faster than women infected with only one HPV type[17], [18]. This synergistic effect could possibly promote condyloma development in patients concurrently infected with non-oncogenic as well as oncogenic HPV types. A simpler explanation could be that infection with oncogenic HPV may be a surrogate marker for infection with or exposure to non-oncogenic HPV types. Prior study of HIV-negative MSM found that 45% of those with anal HPV infections were infected with more than one HPV type, with both oncogenic and non-oncogenic HPV types being most prevalent[16]. It is clear that qHPV vaccination did not achieve a significant reduction in recurrent condyloma in the smaller subset of patients who had been treated and disease free for at least 12 months prior to study entry. The Kaplan-Meier curve for cumulative recurrence, however, does show a striking separation between the vaccinated and non-vaccinated patients beginning at approximately 3 years, with a marked reduction in p-value for the difference between the curves (Figure 2). While the failure to achieve significance in the subset of patients with prior history of condyloma could be related to vaccine failure, it could also be related to the small number of vaccinated patients followed for 3 or more years. A large, prospective, randomized trial would hopefully answer this question. In the women’s qHPV study, Joura et al. noted a 47% decrease in recurrence of genital condyloma related to qHPV types in the women who received qHPV vaccine versus placebo. As with our study, the difference did not reach statistical significance[19].

There are several limitations to our study. First, there were significantly more unvaccinated patients in our cohort and they were followed for longer time overall than were the vaccinated patients. Therefore, our results favoring the protective effects of qHPV might be biased towards the vaccinated patients, as more follow-up time could have revealed additional cases of anal condyloma. However, despite this potential bias, our study suggests that qHPV has the ability to protect older MSM against anal condyloma, which could be studied further in a larger, more systematic trial. Secondly, the vaccinated and unvaccinated groups were not comparable at baseline in several aspects. However, univariate analysis determined that these differences did not significantly affect the outcome enough to be included in the multivariable models. It is possible that unmeasured variables differed between the two groups, as related to who chose to get vaccinated and was able to pay for vaccination. A previous study of patients in this practice who refused qHPV found the most cited reasons for lack of vaccination were not knowing enough about the vaccine, already being infected with HPV, lack of FDA approval, and cost[20].

This non-concurrent cohort study relied on medical records, limiting available data to information contained in the medical chart. Knowing non-oncogenic HPV status for all patients would have greatly enriched our understanding of the potential protective mechanism of qHPV in anal condyloma development. While it would have been beneficial to evaluate the patients’ sexual practices both pre- and post-vaccination, sexual practice was not noted in charts systematically enough to include in statistical modeling. Missing data on race/ethnicity prevented studying its effect on condyloma development and prevented adequate adjustment in the analysis. This was a population of mostly white, urban, non-smoking MSM with private insurance; findings may not be generalizable to other populations. Lastly, we did not have the ability to perform HPV subtyping of lesions to determine if disease in vaccinated patients may have been due to non-qHPV types.

This is the first study to show that qHPV decreases risk of anal condyloma among older MSM. Although the vaccine is currently licensed and recommended for prevention of HPV infection in young persons ages 9 to 26 years, if our results are confirmed by a randomized, placebo-controlled trial the age of the target population should be expanded, especially in high-risk populations like MSM.
